# Personal experience: Coming out - the psychotic psychiatrist - an account of the stigmatising experience of psychiatric illness

**DOI:** 10.1192/pb.bp.113.044016

**Published:** 2014-08

**Authors:** Aashish Tagore

I am proud to be a psychiatrist. Despite often feeling stigmatised by medical colleagues, I have always derived a great sense of personal reward and satisfaction from my chosen line of work. I believe psychiatrists are blessed to be in a position to assist people at times of great personal distress and emotional turmoil, often when they are at their most vulnerable. As psychiatrists, we are only too well aware of the stigma and negative attitudes our patients face in society in general. There can be no doubt that many of our patients with severe mental illnesses are marginalised to the fringes of society, often leading their lives in depressing isolation. They are often ostracised by others, who treat them with contempt. Our patients are often made to feel like ‘freaks’ by an intolerant and ignorant society. They are rejected as pariahs. To some degree, these attitudes are borne out of a fear of the unknown (or at least, poorly understood), and in the case of psychotic patients, a misperception that they are a danger to society.

I am sure that all psychiatrists, like myself, try to have an understanding of how this must feel for our patients and, in so doing, we strive to treat them with compassion, empathy and humanity. I am also sure that the vast majority of us robustly back the plethora of campaigns that aim to destigmatise mental illness in our society. But what happens when you suddenly find yourself on the ‘other side of the fence’? This is the scenario I have recently been faced with, and that has challenged my genuine dedication to the anti-stigma cause. And so it is after a great deal of reflection and soul searching that I have decided to ‘come out,’ and write this account of my own experience of psychotic illness, and more specifically the stigma I experienced along the way.

## How it all began

Last year, I was suspended from work following an allegation of a rather disturbing and sensitive nature (which was later proven to be completely baseless). Needless to say, the entire episode was nothing short of traumatic. The experience was surreal beyond imagination, not least because I had absolutely no clue as to exactly what the allegation referred to. I was not (and still to this date have not) been provided with any more information as to what, when, or where the alleged incident occurred. Perhaps most people presented with a similar set of circumstances would find themselves feeling equally bewildered and bemused. It seems to me, that if one does not have any frame of reference around which to process such an event, an adverse impact on one’s psychological well-being is inevitable. Alas, it was against this backdrop of complete secrecy and a wall of silence, that, perhaps unsurprisingly, conspiracy theories and paranoid ideas began to take over from rational thought. Before I knew it, I was experiencing highly systematised, persecutory delusions, and was convinced that my life was in grave danger. A precipitous descent into full-blown paranoid psychosis ensued, which culminated in a hospital admission and treatment with antipsychotic medication.

## Is openness the right decision?

I have been advised by many people, not least family and close friends, all of whom undoubtedly have my best interests at heart, to remain tight-lipped about both the allegation and my ensuing illness. Perhaps my decision to speak out about my experiences are at least partly driven by a desire to break free from these shackles of secrecy. I feel that other people’s wishes to conceal this whole episode have served to compound and perpetuate my own sense of shame and embarrassment as a result of experiencing a psychotic illness. Perhaps this is a cathartic exercise of candour, designed to enable me to feel liberated from the air of secrecy that has surrounded my illness? Who knows....

Of course, I can understand where they’re coming from. I have no doubt that their main concern is the effect that such openness will have on my future career. They are certain that to openly admit to a psychotic illness would be ‘career suicide’, and that I would never be considered for a senior job. After all, what department would want to hire and work alongside a colleague whose mental stability is a source of constant concern. Surely they would prefer someone dependable and resilient to stress. Of course, I can completely understand this perspective - who would choose to work alongside someone who may end up on protracted periods of sick leave? Who would choose someone who might become paranoid towards them, might start to believe they are being plotted against, or might start behaving bizarrely in some way when at work? Surely it would be easier to simply avoid such potentially awkward situations, and hire someone with a clean bill of mental health? I myself would certainly have thought along these lines. And so did my treating psychiatrist: to some degree, at least. Looking at his correspondence to my employing trust, he clearly attempted to minimise the psychotic quality and severity of my illness, preferring to conceptualise it as a ‘severe adjustment disorder in the context of extreme stress’. He also made a point of explicitly stating that I did not have schizophrenia. There is no doubt in my mind that his objective in euphemising my condition was as a damage limitation exercise for my career prospects. In essence, it appears that many doctors (including psychiatrists), expect or anticipate that there will be inevitable discrimination against fellow medics who have had a mental illness.

## The great leveller

The most difficult time I had to endure during my illness was when I was admitted to a psychiatric unit. Fortunately, I was only admitted for 1 day, but short-lived as it was, the impact it had on me will endure far in to the future. This was when the truth really hit my between the eyes - I was now a bona-fide mental patient. I have never felt such an acute sense of dismay and helplessness as I did at that precise point. Here I was, the newest patient on a ward on which I used to work - it just didn’t seem real. It was my worst nightmare realised. Upon reflection, this period was also when the stigma of my illness came to the fore. Not only was I embarrassed to be there, the staff with whom I used to work alongside appeared equally embarrassed for me. The pity was written all over their faces. And I must confess that my own prejudice towards mentally ill patients surfaced. Despite evidently needing acute assessment and treatment, through my psychotic haze, I still felt I was for some reason better than or at least different to the other patients on the unit. I felt the need to distance myself (both physically and psychologically) from the other patients - I needed to reassure myself that I was not one of them. But, alas, I was. I was no better than or different to them - I was just as unwell, and just as human as the rest of them. I was just as vulnerable and susceptible to mental illness as the rest of them. I was just as breakable as they were. I was no longer this superior being, the ‘doctor’ to their ‘patient,’ I was their equal. Despite the fact that I hated every moment of my admission, it was a great leveller. Any airs and graces I had all but disappeared in the course of that day.

## Self-stigmatisation

In the immediate aftermath of my episode, I felt a sense of deep-seated shame and guilt. I never thought that a life event, traumatic as it was, could have had such an adverse impact on my mental well-being. I felt as if I had let down not only my wife and my family, but also myself. Prior to my illness, I identified with myself as a resilient individual who was able to deal with stress and adversity. However, the illness, perhaps unsurprisingly, knocked my confidence, and with it, my self-belief. I now viewed myself as a ‘weak-minded’ individual.

And so it is with the concept of ‘self-stigmatisation’, where the stigmatised individual actually relates to others’ negative attitudes towards themselves and their illness. If you yourself share the negativity you might endure, and view such attitudes as ‘understandable’, then where is the motivation to fight it? I experienced these feelings until fairly recently following my psychotic illness, and almost felt that I didn’t deserve to be treated the same as everyone else, or indeed, the same as my pre-psychotic self. It’s strange how the stigma of mental illness affects one’s self-identity so profoundly.

Another aspect of feelings of shame in the immediate aftermath of my illness, relates to the behaviours and actions that were integral manifestations of my psychotic experience. For example, at one point I confronted a neighbour because I believed they were spying on me (in fact, I approached the police about this, such was the conviction of my beliefs). Looking back on this incident now, I feel utterly mortified. My sense of embarrassment regarding this particular incident is indescribable. And thus, rather than approach this neighbour and explain that I was mentally unwell at the time, I continue to choose to avoid them. Perhaps I still have some way to go to being at ease and open with my illness after all. That’s stigma for you, I guess. I reflect on this particular incident frequently, and wonder how patients must feel when they have recovered from an acute episode of their illness, but can still recall embarrassing behaviours. Consider those recovering from acute manic episodes in particular, many of whose illnesses are highlighted by extreme, reckless, chaotic and disinhibited behaviour. Their sense of humiliation must be profound, and must linger in their minds long after their mania has subsided.

## Stereotypes and prejudice

My family and friends must also be certain that people will judge me negatively, just as they do my patients, and that I too, will be labelled a ‘freak’ or a ‘nutter’. And as much as they care for my welfare, I guess there is also an element of shame and embarrassment for my family through association. After all, if they were slightly embarrassed to tell their friends that their beloved doctor son had chosen to become a psychiatrist, how would they feel telling their friends that he had also now become a psychiatric patient. How they must have imagined their friends mocking, ‘it’s true what they say isn’t it, it takes one to know one, I told you all psychiatrists were mad themselves!’ After all, it is not an uncommon perception, even among medics, that you either have to be ‘mad’ to do psychiatry, or that exposure to ‘mad’ patients will eventually make you ‘mad’ too. An interesting chicken-and-egg debate among medical students, as I recall from my own university days!^1^

I suppose I’m not doing much to dispel that particular myth. Indeed, many of my most stigmatising experiences came from within the medical profession, as opposed to the wider general public. Most of these incidents were borne out of negative stereotyping of mental illness. The most memorable example came when I met with the medical director of the trust in which I was working, with a view to finding out more about the allegation I faced. During this consultation, it became clear that this very senior doctor believed my circumstances were essentially self-inflicted, commenting that such false accusations must be an ‘occupational hazard’ in psychiatry, and that, as such, I only had myself to blame for choosing the specialty. It became clear that he seriously believed that many psychiatric patients must have a propensity to make deliberately false, malicious allegations, as if this trait is in their nature. I was initially quite taken aback by his prejudiced stereotyped attitude towards psychiatric patients, until I came to realise his opinions were shared by many of my medical friends too. It is this kind of negative stereotyping that contributes hugely to the stigma of mental illness. That is not to say psychiatrists should take the moral high ground over their medical counterparts on this front. Indeed, it seems that we our the most hypocritical when it comes to prejudiced attitudes towards our own patients. And I include myself in this. I wonder how many psychiatrists can honestly say they treat all their patients with equal regard, irrespective of the diagnostic label with which they come attached? I am certain many psychiatrists hold prejudiced and stereotyped preconceptions, and make negative judgements when they are asked to assess a patient with a diagnosis of borderline personality disorder or drug/alcohol misuse. Most would far prefer to see patients with ‘real’ psychiatric illness, such as schizophrenia. What is this, if not an extension of the prejudiced attitudes our medical colleagues display towards psychiatric patients as a whole? Are psychiatrists, then, as supposed advocates of mentally ill people, not guilty of the greatest hypocrisy when it comes to challenging the negative stereotypes associated with mental illness?

## The stigma differential

As far as wider society’s viewpoint of mental illness is concerned, there seems to be a differential degree of stigma and prejudice attached to different mental disorders. Depression seems to be more widely understood and accepted these days, and people with depression seem more at ease in disclosing their illness. Additionally, the ‘understandability’ of depression, particularly as a reaction to adverse life events, makes it a more palatable proposition to others. By contrast, it seems that psychotic illnesses have a much greater stigma attached to them, partly due to persisting misconceptions (for example dangerous and violent people), and partly because they lack the ‘understandability’ of depressive illnesses, in that their symptoms are completely alien concepts for many people. Thus, people find it more difficult to put themselves in your shoes.

I experienced this differential stigma at play when it came to being open about my own illness. I have no doubt that I would have found it far easier to disclose to people that I had depression as a result of an extremely stressful period, as opposed to a psychotic episode. However, although I would have been more at ease admitting to depression, I am not so sure that I would have received as sympathetic a reaction from most. In the case of depression, many people, although being able to ‘relate’ to the condition in such circumstances, will have underlying emotions that the individual needs to ‘snap out of it’, or ‘pull themselves together’ or ‘get a grip’. They will view this reaction as a sign of weakness and self-pity in the individual, which will in turn elicit feelings of annoyance or irritation, rather than sympathy. Conversely, although the psychotic experience will be far removed from ‘normal’ experiences, people would be less inclined to view it as a character flaw, and more as a genuine (albeit alien) ‘disease’. They may also perceive it to be less under the control of the individual, and thus may be more sympathetic to them.

This differential stigma also extends to my own willingness to open up about my illness. On the other hand, I think that I have only been able to open up about my illness because it occurred within the context of an extremely distressing situation, and because it seems to have been a transient phenomenon. I have no doubt that had I been diagnosed with a chronic psychotic illness (such as schizophrenia), which did not occur in the context of severe stress, and that had not abated completely, I would not have been able to write this article. Furthermore, had this been the case, I have no doubt that people (friends and family included) would be treating me very differently: with greater caution, wariness and a persisting sense of disconnection and estrangement towards me. It is clear that they have only been able to accept my illness because it was short lived and transitory, a thing of the past, which can be resigned to history: ‘As long as you’re back to normal now, that’s the main thing’.

## Accepting a mental health diagnosis

So what have I learnt from my experience of psychosis and the stigma that comes as part of the package with such an illness? Looking back now, the experience felt like the classical stages of the grieving process, as described by Prochaska and DiClemente. For a period of time in the aftermath of my illness, I went through stages of denial, anger, bargaining and depression, and it seems as though the stigma attached to the illness contributed to these stages more than the actual experience of mental illness itself. As the title of a recent article in the Independent newspaper eloquently puts it, ‘the stigma of mental ill health is worse than the illness’. Ultimately, achieving a state of acceptance of one’s illness in reality means learning to overcome the stigma that is attached to it. For only then is one able to embrace the illness, process it positively, and move on with one’s life with some degree of confidence. Based on my own experience of psychotic illness, it seems clear to me that the many people who have a mental illness would have a far better prognosis if they didn’t have to deal with the associated stigma and negative attitudes from society at large. For it is this aspect of mental illness that proved the most difficult to overcome. Long after the acute psychotic symptoms have abated, it is the stigma that is the residual source of persisting distress, and functional impairment. For stigma has the power to irrevocably destroy one’s sense of self-worth, and to grossly distort one’s self-identity.

## Concerns for the future

I am relieved to say that I am enjoying a period of sustained mental stability, that I can only hope continues. Interestingly, the issues that might impede my recovery to full functioning seem to relate to issues of stigma. For example, I have recently returned to work on a phased basis. It is with some trepidation that I have resumed my training, perhaps unsurprisingly, after such a protracted period of sickness. Prior to returning, I had a long discussion with my clinical supervisor about the possibility of becoming unwell again while at work. How would this be picked up at an early stage, so as to minimise the distress to myself and, of course, to avert any potential for patient harm. It was something that I had hitherto not given much consideration. I had to admit to her that I may well find it difficult to actually openly disclose any paranoid ideas or other thoughts that may be suggestive of a relapse (working on the massive presumption that I would be able to identify such thoughts as symptoms of illness in the first place!). I tried to imagine how I would actually go about informing my supervisor that I was experiencing ‘abnormal’ thoughts, if such an eventuality arose. It was at this point that I realised admitting to such ‘symptoms’ continues to be a source of embarrassment to me. The thought of approaching my consultant and saying something along the lines of, ‘Hi there, I think I might be developing paranoid ideas, and I think I’m hearing voices’, makes me cringe to my core. But why? If I had returned to work after a long lay-off following a bout of physical illness (for example heart problems), would I have any issues informing her that I was experiencing chest pain? Of course not. So what is the difference? It comes down to the shame and/or embarrassment of admitting to mental ill health as compared with physical, and this is another facet of the stigma associated with such illness. And so we can see that if such issues around stigma impede one’s ability to seek help in a timely manner, surely they could potentially have an adverse impact on one’s future prognosis. And so it must be for many of our patients - they don’t seek help proactively because of feelings of shame and embarrassment regarding their illness, as opposed to deliberately disengaging because of a poor therapeutic alliance with their team, or lack of insight in to their illness. For myself, at least, I have realised that I will always find it difficult to openly admit to symptoms suggestive of a relapse, purely as a result of the way I perceive others will view my illness.

Stigma can rear its ugly head in the most subtle of ways, even in situations where people mean no ill will. When I go to the pharmacy to collect my prescription, I notice the chemist’s double-take at the list of medications, followed by a sly glance at me and a whispered conversation with their colleague. Of course, they mean nothing by this, but one can’t help wondering what they are thinking about you. I do not recall the same pharmacists batting an eyelid when the only medications I was prescribed were lansoprazole and ferrous sulphate - no stigma attached to indigestion or anaemia, then. Such changes in behaviour towards oneself aren’t particularly upsetting in and of themselves, but they do contribute to the general air of negativity one feels when living with mental illness. You just know that people view you differently than they did before.

So, you may ask, why did I come to the decision to share my experience of psychotic illness, particularly in the face of advice to the contrary from so many quarters. At the end of the day, I surmised, all my anti-stigma support for my patients wouldn’t mean a thing, unless I was willing to practise as I preach, and to put my money where my mouth is. After all, what kind of a psychiatrist would I be if, after preaching to my own patients not to be ashamed or embarrassed about their conditions, I chose to conceal my own illness? To my mind, this would make me nothing short of a hypocrite. I would like to think this decision came in the form of an enlightened ‘Eureka’ moment, but alas, this is not the case. Rather, it has been a constant struggle with mixed and ever-changing emotions, and I still don’t know how it will affect my future. It is very likely that potential employers and concerned relatives will view this as a foolhardy exercise, rather than one of personal dedication to the cause of destigmatising mental illness. However, should that be the case, I have concluded that this would reflect negatively on them, and not me.

So now that I have learnt to accept my psychotic illness, I hope that I may be able to use it to my advantage in my clinical practice. I genuinely believe the experience has improved my capacity to understand what my patients are experiencing on a much more personal level. In essence, I would like to think it will help me become a better psychiatrist. Like mental illness, the stigma that comes with it is a multifaceted and complex concept. From my own personal experience, the stigma associated with mental illness is just as debilitating as the symptoms. For although most patients will usually get some (albeit temporary or partial) relief from their undoubtedly distressing symptoms, there is no such reprieve with stigma - it is unrelenting in its negative impact. Stigma has the power to smother you with its secrecy and prejudice. The word may be used metaphorically in the context of mental illness, but it sometimes makes you feel as if you are literally marked or stained.
